# County‐level colorectal cancer screening rates on colorectal cancer survival in the state of Georgia: Does county‐level rurality matter?

**DOI:** 10.1002/cam4.6830

**Published:** 2024-01-02

**Authors:** Meng‐Han Tsai, Steven S. Coughlin, Jorge Cortes

**Affiliations:** ^1^ Cancer Prevention, Control, & Population Health Program, Georgia Cancer Center Augusta University Augusta Georgia USA; ^2^ Georgia Prevention Institute Augusta University Augusta Georgia USA; ^3^ Department of Biostatistics, Data Science and Epidemiology Augusta University Augusta Georgia USA; ^4^ Georgia Cancer Center Augusta University Augusta Georgia USA

**Keywords:** colorectal cancer survival, county‐level colorectal cancer screening, county‐level rurality, Georgia

## Abstract

**Purpose:**

Investigating CRC screening rates and rurality at the county‐level may explain disparities in CRC survival in Georgia. Although a few studies examined the relationship of CRC screening rates, rurality, and/or CRC outcomes, they either used an ecological study design or focused on the larger population.

**Methods:**

We conducted a retrospective analysis utilizing data from the 2004–2010 Surveillance, Epidemiology, and End Results Program. The 2013 United States Department of Agriculture rural–urban continuum codes and 2004–2010 National Cancer Institute small‐area estimates for screening behaviors were used to identify county‐level rurality and CRC screening rates. Kaplan–Meier method and Cox proportional hazard regression were performed.

**Results:**

Among 22,160 CRC patients, 5‐year CRC survival rates were lower among CRC patients living in low screening areas in comparison with intermediate/high areas (69.1% vs. 71.6% /71.3%; *p*‐value = 0.030). Patients living in rural high‐screening areas also had lower survival rates compared to non‐rural areas (68.2% vs. 71.8%; *p*‐value = 0.009). Our multivariable analysis demonstrated that patients living in intermediate (HR, 0.91; 95% CI, 0.85–0.98) and high‐screening (HR, 0.92; 95% CI, 0.85–0.99) areas were at 8%–9% reduced risk of CRC death. Further, non‐rural CRC patients living in intermediate and high CRC screening areas were 9% (HR, 0.91; 95% CI, 0.83–0.99) and 10% (HR, 0.90; 95% CI, 0.82–0.99) less likely to die from CRC.

**Conclusions:**

Lower 5‐year survival rates were observed in low screening and rural high‐screening areas. Living in intermediate/high CRC screening areas was negatively associated with the risk of CRC death. Particularly, non‐rural patients living in intermediate/high‐screening areas were 8%–9% less likely to die from CRC. Targeted CRC screening resources should be prioritized for low screening and rural communities.

## 1 INTRODUCTION

Colorectal cancer (CRC) is the one of most common cancer death among men and women in Georgia with 14.1 estimated deaths per 100,000 populations, which is higher than the rates in the United States (US) (13.1 per 100,000) in 2020.^1^, ^2^ Despite this higher burden in mortality, there has been an average reduction in annual mortality rate of 2.3% per year during the period from 2002 to 2013.^1^ Systematic CRC screening efforts and better treatment have contributed to these reduced trends in Georgia.^3^ According to the U.S. Preventive Services Task Force (USPSTF) and American Cancer Society (ACS) recommendations on CRC screening, average‐risk individuals should have routine screening to reduce CRC mortality.^4^, ^5^ Regular CRC screening with either a high‐sensitivity stool‐based test or a visualization test (e.g., colonoscopy or sigmoidoscopy) are recommended for screening eligible individuals.^5^ In Georgia, there was an increase in the utilization of colonoscopy or sigmoidoscopy for CRC screening between 1997 and 2018, with rates increasing from 48.1% to 71.2%.^3^ Despite these improved rates, screening use in Georgia is still behind the national goals.^6^

While CRC mortality has improved over time, these improvements have not been equally distributed across different geographic areas. Evidence has shown that decreased access to health care, including cancer screening, was positively associated with poorer cancer outcomes for CRC patients.^7^ Barriers contributing to the accessibility of cancer screening may be due to limited screening facilities and lack of transportation.^8^ These barriers have been strongly tied to the place where patients live. In particular, counties designated as rural areas commonly face several challenges including systematic disinvestment and hospital closures that may greatly impact rural residents' overall health.^9^ Remarkably, CRC mortality tends to be higher in rural than non‐rural counties, with a 16%–22% increased risk of CRC death among rural patients.^10^, ^11^ Therefore, investigating CRC screening rates and rurality at the county‐level may have the potential to explain disparities in CRC survival in Georgia.

Although a few studies examined the relationship of CRC screening rates and CRC outcomes, they either used an ecological study design but without considering any inferences at the individual‐level information^12^, ^13^ or focused on individual‐level data only.^14^ Moreover, limited studies have also examined the impact of rurality on CRC outcomes within the small areas.^15^, ^16^ A study that examines these relationships within small geographic areas by using county‐ and individual‐level information is critical because it may inform local policies for ongoing investment in low health related resource communities. Because there are 124 out of 159 counties in Georgia are designated as rural by the Georgia Rural Development Council,^17^ Georgia is particularly suited for the study of these relationships among underserved populations. To address the research gap, we sought to (1) examine 5‐year CRC survival rates according to county‐level CRC screening rates and stratified by county‐level rurality, and (2) examine the association between county‐level CRC screening rates, county‐level rurality, and 5‐year CRC survival in Georgia.

## 2 METHODS

### 2.1 Study design

This is a retrospective cohort study utilizing county‐level and individual‐level information from publicly available databases. For county‐level data, we used data from the 2013 United States Department of Agriculture rural–urban continuum codes (RUCC)^18^ and the 2004–2010 National Cancer Institute (NCI) small‐area estimates for cancer risk factors and screening behaviors.^19^ Individual‐level data from the 1975–2016 Surveillance, Epidemiology, and End Results (SEER) Program were used, which is a source for comprehensive population‐based information in the US that includes patient demographics, primary tumor site, tumor morphology and stage at diagnosis, first course of treatment, and follow‐up for vital status. Further, we used the county Federal Information Processing System (FIPS) code as identifiers to link these three databases for CRC patients in Georgia.^20^ Data sources extracted for this study were publicly available and de‐identified, and thus considered exempt from Institutional Review Board (IRB) review.

### 2.2 Study participants

The study eligible population included patients diagnosed with CRC defined by the SEER Site Recode ICD‐O‐3/WHO 2008 definition of colon cancer (C180–C189), rectosigmoid junction cancer (C199), and rectal cancer (C209).^20^, ^21^ A total of 997,685 CRC patients were included in 1975–2016 SEER 18 registries custom data, November 2018 submission (http://seer.cancer.gov). Initially, we excluded CRC patients aged less than 18 years (*n* = 675), as well as those with repeated diagnosis of CRC (*n* = 44,809), missing rural and non‐rural information (*n* = 14,109), CRC diagnosed after 2011 (due to their limited follow‐up time of less than 5 years) (*n* = 177,707), and missing survival time (*n* = 9681). To obtain an eligible study sample in Georgia, we further excluded patients who did not live in Georgia (*n* = 689,736), missing cancer sites (*n* = 343), were diagnosed with CRC before 2004 or after 2010 (*n* = 35,154) and were aged <50 years (*n* = 3311). The selection criteria of diagnostic year and age were based on the National Cancer Institute (NCI) small‐area estimates of county‐level cancer screening behaviors, which were estimated using the 2004–2010 Behavioral Risk Factor Surveillance System (BRFSS) and National Health Interview Survey (NHIS) among adults aged 50 + years.^19^ As a result, 22,160 patients diagnosed with CRC during 2004–2010 and living within Georgia were included as the final sample for statistical analysis (Figure S1).

### 2.3 Outcome and exposures of interest

CRC survival was our outcome of interest. Our primary exposure of interest was county‐level CRC screening rates. For CRC screening rates, we used data from the National Cancer Institute (NCI) small‐area estimates of county‐level cancer screening behaviors, which reflect actual use of cancer screening. We gathered the 2004–2007 and 2008–2010 prevalence estimate for CRC screening use by using CRC test ever section.^19^ The definition of CRC test ever is that an individual ≥50 years must have reported having at least one colorectal endoscopy (sigmoidoscopy or colonoscopy) in their life or at least one home‐based Fecal Occult Blood Test (FOBT) within the past 2 years by the time of interview. Finally, we classified county‐level CRC screening rates as a three‐level variable (low, intermediate, or high) by using common quantiles (5 quantiles) based on the NCI small‐area estimates as cut‐off points.^19^ Common quantiles are calculated by polling all years of data for the same outcome, geography, and sex groupings.^19^ Finally, we defined counties with CRC screening rates ≤45.4 (lowest quantile) as low, 45.5–60.7 (middle three quantiles) as intermediate, and ≥60.8 (highest quantile) as high.

County‐level rurality was our second exposure of interest. Data from the 2013 United States Department of Agriculture rural–urban continuum codes (RUCC) were used to define rurality. The 2013 RUCCs form a classification scheme that distinguishes metropolitan counties by the population size of their metro area, and nonmetropolitan counties by degree of urbanization and adjacency to a metro area.^18^ Georgia counties with RUCCs of 1–3 are metropolitan (non‐rural), and counties with RUCCs of 4–9 are nonmetropolitan (rural). Finally, we defined county‐level rurality as yes or no.^18^

### 2.4 Covariates

Individual‐level covariates including demographic characteristics and tumor features were adjusted for their impact on the association between county‐level CRC screening rates, county‐level rurality, and CRC survival in Georgia. In demographic characteristics, we included age at diagnosis (50–74 or 75+ years), gender (male or female), marital status (single, married, others, or unknown), and Georgia regions (north, south, central, or other regions). Georgia counties were defined based on the 10 Georgia public health districts by using FIPS codes.^1^ North region includes Northeast, North Georgia, North, and Northeast districts; South region includes South, Southwest, Coastal, and Southeast districts; Central region includes South Central, North Central, East Central, and West Central district; Other region includes Cobb–Douglas, Fulton, Clayton, East Metro, DeKalb, and LaGrange districts.^1^ Further, in tumor features, we included grade (grades 1, 2, 3 & 4, or unknown), stage at diagnosis (localized, regionalized, distant, or unknown), and primary site (right or left).

### 2.5 Statistical analysis

Descriptive statistics were used to describe county‐level CRC screening rates, county‐level rurality, demographic characteristics, and tumor features. We also examined bivariate differences within county‐level CRC screening rates (low, intermediate, or high) in county‐level rurality, demographic characteristics, and tumor features, using chi‐square tests. Patients' survival time was measured in months from the date of diagnosis up to 60 months of follow‐up, censored at the end of the study observation period (December 31, 2016), or death. Survival analyses at 5‐year intervals were applied using the Kaplan–Meier method. The Log‐rank test was performed to compare the survival rates within county‐level CRC screening rates and stratified by county‐level rurality. Further, we performed Cox proportional hazard regression to examine the impact of county‐level CRC screening rates on CRC survival among Georgians. Four sequential models were performed to examine the association. The crude model included county‐level CRC screening rates only; model 1 was adjusted for county‐level rurality; model 2 was further adjusted for demographic characteristics; and model 3 was further adjusted for tumor features. Finally, we examined the interaction between county‐level CRC screening rates and county‐level rurality on CRC survival. Such modification enables the explanation on whether county‐level rurality impact the association between county‐level CRC screening rates and the risk of CRC death. The effect modification model was adjusted for individual‐level covariates (demographic characteristics and tumor features). All results were reported using hazard ratios (HRs) and the associated 95% confidence intervals (CIs). The SAS Version 9.4, SAS Institute Inc., Cary, North Carolina was used to conduct all analyses. The level of statistical significance was set at an alpha level of 0.05, and the *p*‐values were based on two‐sided probability tests.

## 3 RESULTS

### 3.1 Characteristics

As shown in Table 1, 57.8% of CRC patients lived in intermediate screening areas. The majority of patients were in non‐rural areas (77% vs. rural: 23%). Many patients were aged 50–74 years (69.5%), male (51.7%), White (70.5%), married (52.7%), lived in of the region of Georgia classified as “Other” (40%), had grade 2 disease (60.2%), had localized CRC (42.5%), and had left‐sided CRC (58.2%). Features correlated with CRC screening rates at *p*‐values <0.001 included county‐level rurality, race, marital status, and Georgia regions. A majority of non‐rural patients lived in the high CRC screening areas (87.5%) and rural patients were more likely to live in low screening areas (32.8%). Further, the majority of White patients lived in intermediate screening areas (73.2%) while many Black patients lived in low screening areas (38.1%). For marital status, CRC patients living in low screening areas were more often single, divorced, separated, or widowed. Finally, many patients residing in central (30%) and other (46.4%) regions of Georgia had low CRC screening rates, while many of those in North regions (30.4%) had intermediate screening rates, and many of those in South regions (21.6%) had high‐screening rates.

**TABLE 1:** Distribution of rurality, demographic characteristics, and tumor features by county‐level CRC screening rates.

	Total (*n* = 22,160)	CRC screening Low (*n* = 3024, 13.6%)	CRC screening Intermediate (*n* = 12,804, 57.8%)	CRC screening High (*n* = 6332, 28.6%)	*p*‐value
	*n* (%)				
Rurality					<0.001
No	17,072 (77.0%)	2033 (67.2%)	9496 (74.2%)	5543 (87.5%)	
Yes	5088 (23.0%)	991 (32.8%)	3308 (25.8%)	789 (15.5%)	
Demographic characteristics					
Age					0.497
50–74	15,396 (69.5%)	2117 (70.0%)	8914 (69.6%)	4365 (68.9%)	
75+	6764 (30.5%)	907 (30.0%)	3890 (30.4%)	1967 (31.1%)	
Gender					0.425
Male	11,452 (51.7%)	1547 (51.2%)	6590 (51.5%)	3315 (52.4%)	
Female	10,708 (48.3%)	1477 (48.8%)	6214 (48.5%)	3017 (47.7%)	
Race					<0.001
White	15,623 (70.5%)	1831 (60.6%)	9378 (73.2%)	4414 (69.7%)	
Black	6174 (27.9%)	1153 (38.1%)	3252 (25.4%)	1769 (27.9%)	
Other	363 (1.6%)	40 (1.3%)	174 (1.4%)	149 (2.4%)	
Marital status					<0.001
Single	2477 (11.2%)	413 (13.7%)	1358 (10.6%)	706 (11.2%)	
Married	11,669 (52.7%)	1492 (49.3%)	6876 (53.7%)	3301 (52.1%)	
Others^a^	6885 (31.1%)	984 (32.5%)	3951 (30.9%)	1950 (30.8%)	
Unknown	1129 (5.1%)	135 (4.5%)	619 (4.8%)	375 (5.9%)	
Georgia regions					<0.001
North	5151 (23.2%)	243 (8.0%)	3890 (30.4%)	1018 (16.1%)	
South	3929 (17.7%)	469 (15.5%)	2091 (16.3%)	1369 (21.6%)	
Central	4225 (19.1%)	908 (30.0%)	2255 (17.6%)	1062 (16.8%)	
Others	8855 (40.0%)	1404 (46.4%)	4568 (35.7%)	2883 (45.5%)	
Tumor features					
Grade^b^					0.127
Grade 1	2262 (10.2%)	288 (9.5%)	1344 (10.5%)	630 (10.0%)	
Grade 2	13,334 (60.2%)	1849 (61.1%)	7624 (59.5%)	3861 (61.0%)	
Grade 3 &4	3119 (14.1%)	435 (14.4%)	1844 (14.4%)	840 (13.3%)	
Unknown	3445 (15.6%)	452 (15.0%)	1992 (15.6%)	1001 (15.8%)	
Stage					0.323
Localized	9422 (42.5%)	1320 (43.7%)	5451 (42.6%)	2651 (41.9%)	
Regionalized	7503 (33.9%)	988 (32.7%)	4373 (34.2%)	2142 (33.8%)	
Distant	4351 (19.6%)	608 (20.1%)	2469 (19.3%)	1274 (20.1%)	
Unknown	884 (4.0%)	108 (3.6%)	511 (4.0%)	265 (4.2%)	
Primary site^c^					0.080
Right	9272 (41.8%)	1264 (41.8%)	5286 (41.3%)	2722 (43.0%)	
Left	12,888 (58.2%)	1760 (58.2%)	7518 (58.7%)	3610 (57.1%)	

Abbreviation: CRC, colorectal cancer.

^a^ Others include divorced, separated, and widow.

^b^ Grade 1: well differentiated; Grade 2: moderately differentiated; Grade 3: poorly differentiated; Grade 4: undifferentiated.

^c^ Right: cecum to transverse; left: splenic flexure to rectum.

### 3.2 Five‐year survival

**FIGURE 1:**
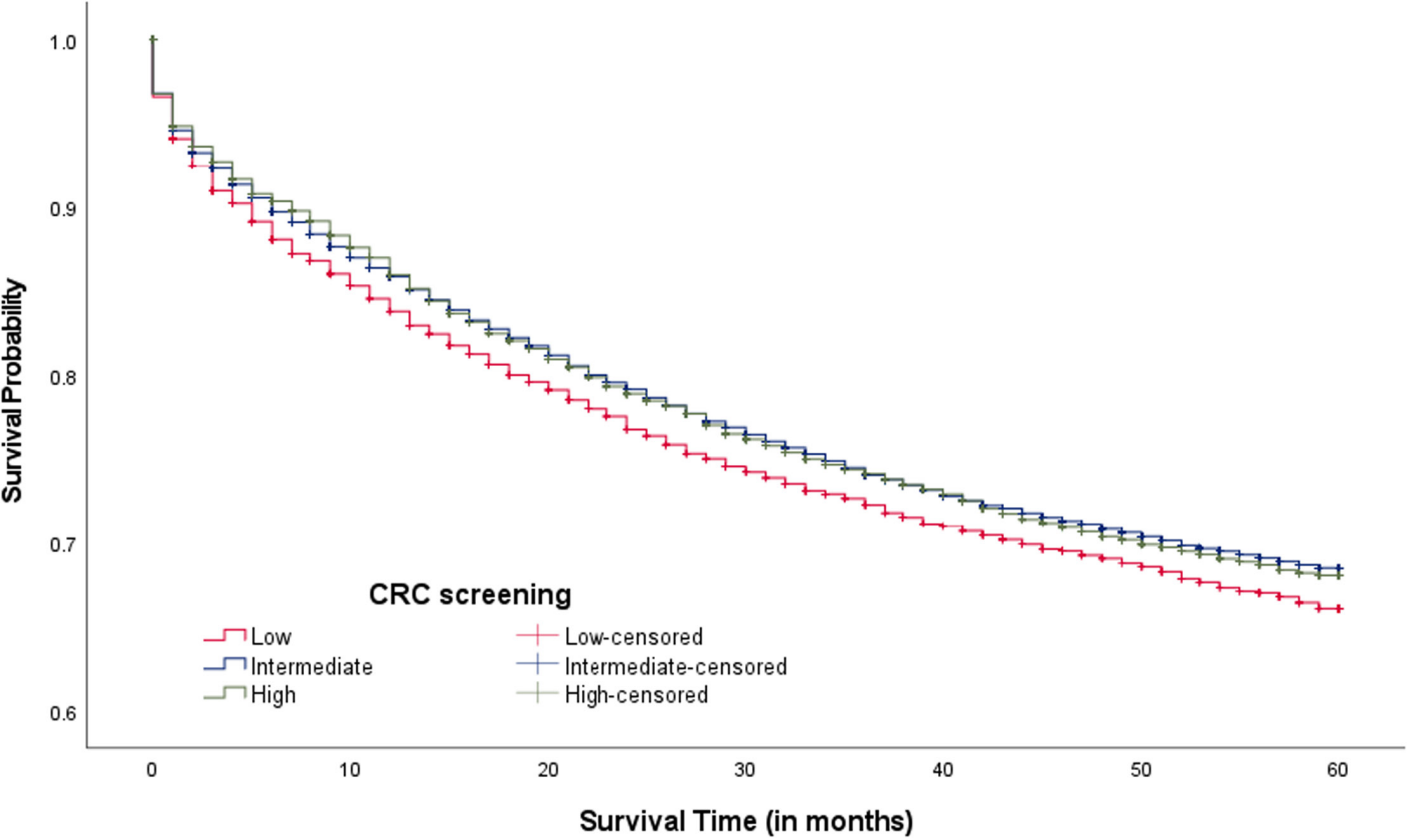
Kaplan–Meier CRC survival curves by county‐level CRC screening rates (*p*‐value = 0.030). CRC, colorectal cancer. Log‐rank test was used.

**FIGURE 2:**
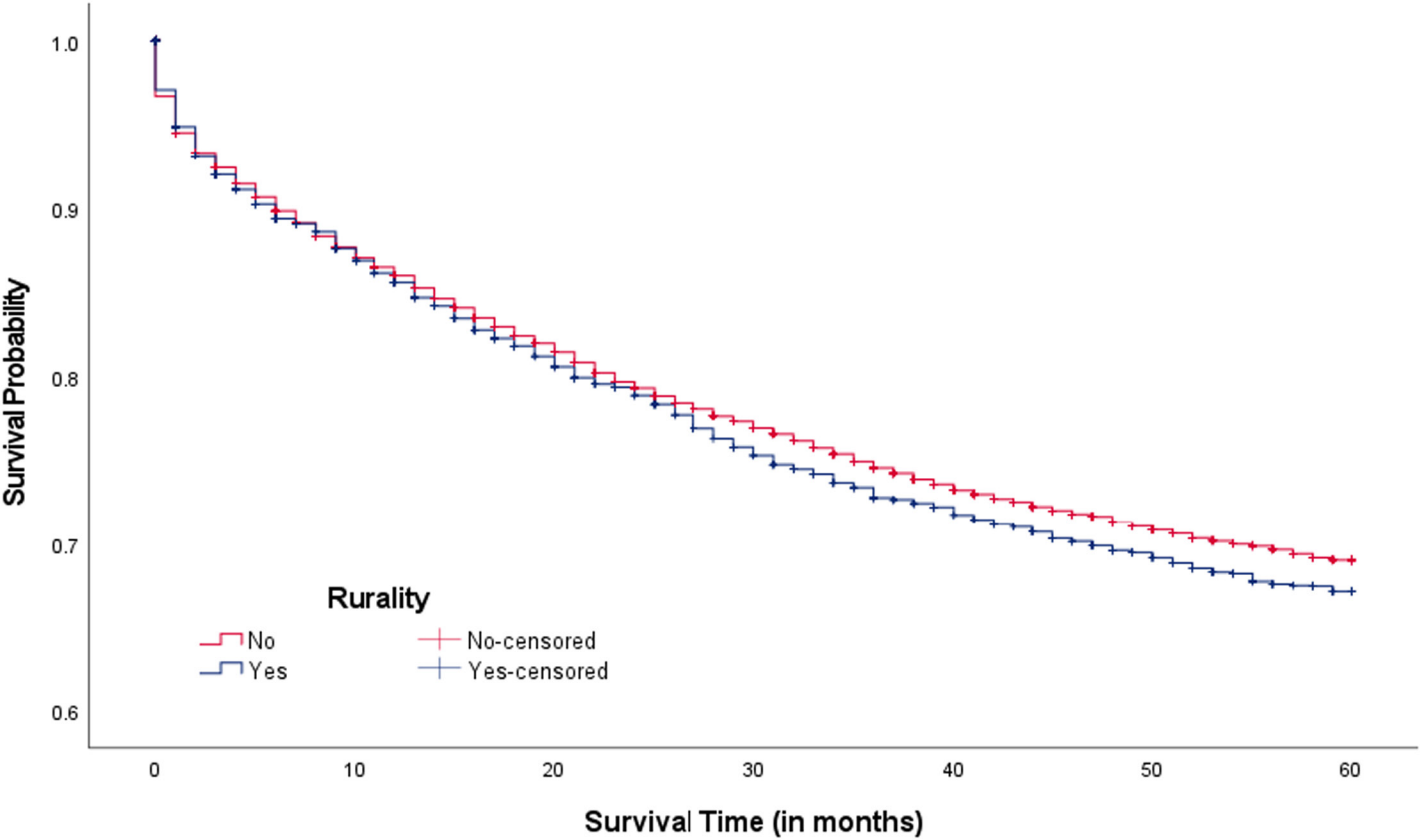
Kaplan–Meier CRC survival curves in intermediate CRC screening areas by rurality (*p*‐value = 0.084). CRC, colorectal cancer. Log‐rank test was used.

**FIGURE 3:**
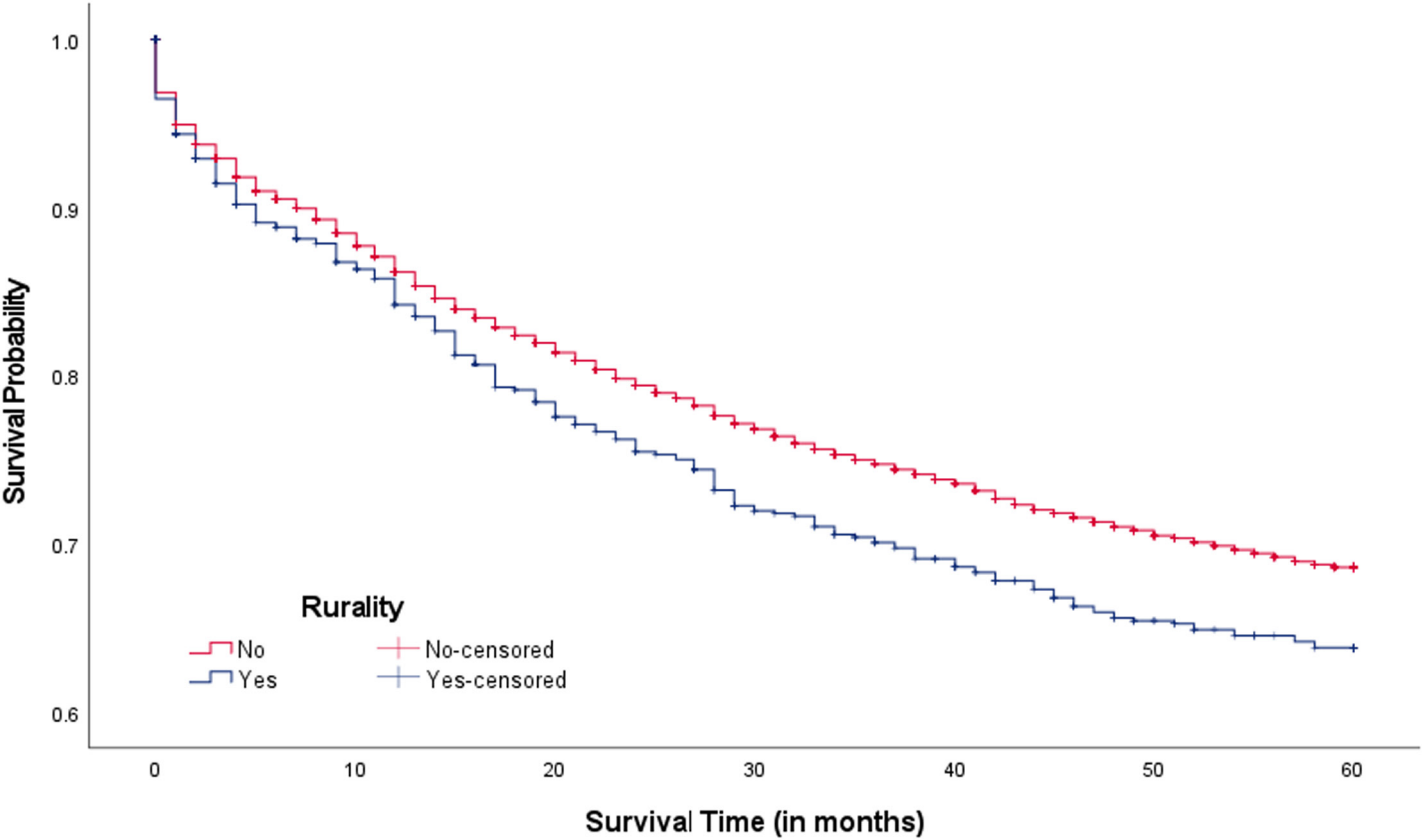
Kaplan–Meier CRC survival curves in high CRC screening areas by rurality (*p*‐value = 0.009). CRC, colorectal cancer. Log‐rank test was used.

**FIGURE 4:**
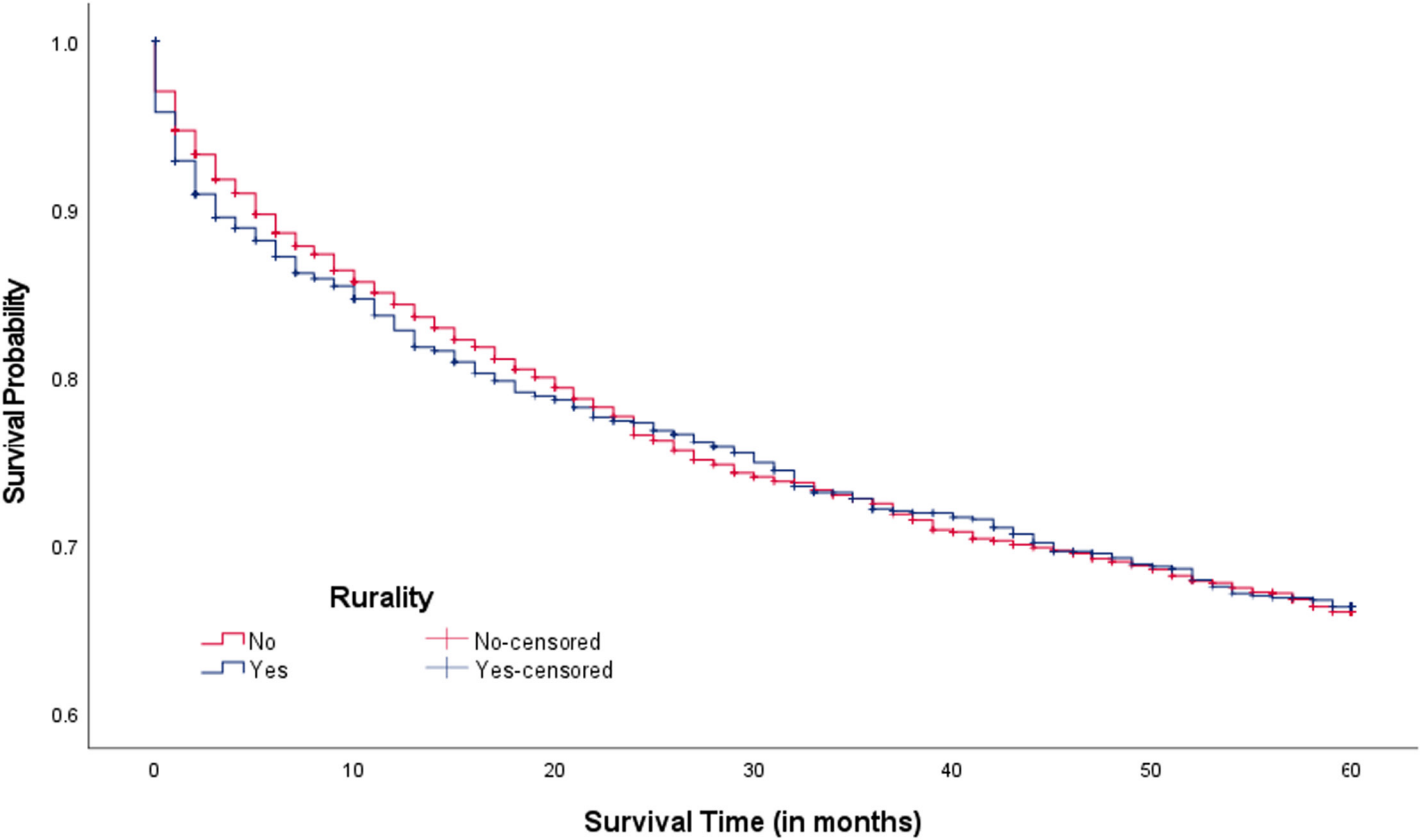
Kaplan–Meier CRC survival curves in low CRC screening areas by rurality (*p*‐value = 0.945). CRC, colorectal cancer. Log‐rank test was used.

Overall, the median survival time since CRC diagnosis was 64.0 months with a range of 0–155 months. In Figure 1, we compared 5‐year CRC survival rates between three CRC screening areas. The 5‐year CRC survival rates were lower among CRC patients living in low screening areas compared to intermediate and high areas (69.1% vs. 71.6% & 71.3%; *p*‐value = 0.030). When examining survival rates among patients living in intermediate and high CRC screening areas within county‐level rurality, those living in rural areas appeared to report lower 5‐year survival rates compared to those not living in rural areas (70.6% vs. 72.0%, *p*‐value = 0.084; Figure 2; 68.2% vs. 71.8%, *p*‐value = 0.009; Figure 3), respectively. Significant differences in 5‐year survival rates were not found in low screening areas according to county‐level rurality (Figure 4).

### 3.3 Association between county‐level CRC screening, county‐level rurality, and CRC survival

Table 2 examines the association between county‐level CRC screening rates, county‐level rurality, and CRC survival adjusted for demographic characteristics and tumor features. Results from each model were slightly different. In the crude model, we found that CRC patients living in intermediate and high‐screening areas were 9% (HR, 0.91; 95% CI, 0.85–0.98) and 8% (HR, 0.92; 95% CI, 0.85–0.99) less likely to die from CRC when comparing to the low screening group, respectively. In models 1 and 2, significant differences of county‐level CRC screening rates on the risk of CRC death almost disappeared when we included county‐level rurality and further adjusted for demographic characteristics in the models. Further, in the full model, similar results were found as in the crude model. Finally, patients living in rural areas had an increased risk of CRC death compared to those not living in rural areas, with a range of 1.08–1.09‐fold increased risk (all *p*‐values <0.05) regardless of covariates adjusted for.

**TABLE 2:** Association between county‐level CRC screening rates, rurality, and the risk of CRC death in Georgia.

	Crude^a^		Model 1^a^		Model 2^a^		Model 3^a^	
	HR (95% CI)	*p*‐Value	HR (95% CI)	*p*‐Value	HR (95% CI)	*p*‐Value	HR (95% CI)	*p*‐Value
CRC screening		0.036		0.052		0.432		0.045
Low	Reference		Reference		Reference		Reference	
Intermediate	*0.91 (0.85,0.98)*		*0.92 (0.85,0.98)*		0.96 (0.89,1.04)		*0.91 (0.85,0.98)*	
High	*0.92 (0.85,0.99)*		0.94 (0.86,1.01)		0.95 (0.89,1.03)		*0.92 (0.85,0.99)*	
Rurality				0.012		0.004		0.010
No	NA		Reference		Reference		Reference	
Yes	NA		*1.08 (1.02,1.14)*		*1.09 (1.03,1.16)*		*1.08 (1.02,1.15)*	

*Note*: Italicized text indicates statistically significant result.

Abbreviations: CRC, colorectal cancer; HR, hazard ratio; NA, non‐applicable.

^a^ Crude model includes county‐level CRC screening only. Model 1 was further adjusted for county‐level rurality; model 2 was further adjusted for demographic characteristics; model 3 was further adjusted for tumor features.

In Table 3, we examined the effect modification of county‐level rurality on the association between county‐level CRC screening rates and CRC survival. Among patients living in non‐rural areas, CRC patients living in intermediate and high CRC screening areas were 9% (HR, 0.91; 95% CI, 0.83–0.99) and 10% (HR, 0.90; 95% CI, 0.82–0.99) less likely to die from CRC compared to those living in low screening areas, respectively. However, statistical significance was not observed within the levels of CRC screening rates among patients living in rural areas (all *p*‐values >0.05). The impact of county‐level rurality on CRC survival was observed among patients living in high‐screening areas (*p*‐value = 0.018). CRC patients living in rural areas and high‐screening areas had a 1.2‐fold increased risk of CRC death when comparing to those living in non‐rural areas and high‐screening areas (HR, 1.18; 95% CI, 1.03–1.34).

**TABLE 3:** Effect modification of rurality on the association between county‐level CRC screening rates and the risk of CRC death in Georgia.

	Rurality no		Rurality yes		
	*n* (%) no/yes 5‐year CRC survival	HR (95% CI)^a^/*p*‐value	*n* (%) no/yes 5‐year CRC survival	HR (95% CI)^a^/*p*‐value	HR (95% CI) for rurality within strata of CRC screening/*p*‐value
CRC screening					
Low (*n* = 3024)	631 (20.9%)/1402 (46.4%)	Reference	303 (10.0%)/688 (22.8%)	1.04 (0.91,1.20)/0.539	1.04 (0.91,1.20)/0.539
Intermediate (*n* = 12,804)	2664 (20.1%)/6832 (53.4%)	0.91 (0.83,0.99)/0.028	973 (7.6%)/2335 (18.2%)	0.96 (0.87,1.07)/0.481	1.06 (0.99,1.14)/0.100
High (*n* = 6332)	1565 (24.7%)/3978 (62.8%)	0.90 (0.82,0.99)/0.025	251 (4.0%)/538 (8.5%)	1.06 (0.91,1.22)/0.461	1.18 (1.03,1.34)/0.018

*Note*: Italicized text indicates statistically significant result.

Abbreviations: CRC, colorectal cancer; HR, hazard ratio.

^a^ Models were adjusted for demographic characteristics (age, gender, marital status, Georgia regions) and tumor features (grade, stage, primary site).

## 4 DISCUSSION

This is the first study to examine the association between county‐level CRC screening rates and CRC survival and consider the effect modification of county‐level rurality on this association in Georgia. Results from our study indicated that 5‐year CRC survival rates were low among patients living in low screening areas and high/rural screening areas. Non‐rural patients living in intermediate and high‐screening areas were at reduced risk of CRC death. Early detection and timely treatment strategies could potentially mitigate the risk of CRC death in low screening and rural areas in Georgia.

### 4.1 5‐year CRC survival rates

When comparing CRC survival rates within levels of CRC screening rates, we found that 5‐year survival rates were 69.1% for those who lived in low screening areas which is lower than patients living in intermediate and high‐screening areas. Significantly lower survival rates were found among those living in rural areas compared to those not living in rural areas (68.2% vs. 71.8%), among those living in intermediate/high‐screening areas. This is consistent with a prior study that suggested more than 50% of the decline in CRC mortality can be attributed to the increased screening utilization.^22^, ^23^ However, rural patients experience a disproportionate level of potentially preventable CRC death when compared with their metropolitan or non‐rural counterparts,^24^ which is also in line with our findings.

### 4.2 County‐level CRC screening rates, rurality, and CRC survival

In our multivariable analysis, patients living in intermediate and high‐screening areas were 8%–9% less likely to die from CRC. These findings may be due to the timeliness and quality of CRC treatment received by patients living in intermediate and high‐screening areas. Living in intermediate and high‐screening areas, with higher availability of CRC screening, may be associated with more sources of care (e.g., treatment options).^25^ Although stage‐at‐diagnosis also influences CRC survival, no significant differences were found in CRC stage‐at‐diagnosis according to whether CRC screening was low, intermediate, or high in our analysis. More research integrating multifaceted factors may further elucidate this relationship. Despite this finding, our study is in line with prior literature on the impact of county‐level CRC screening rates. Yoshida et al. (2018) found that county‐level CRC screening rates were negatively associated with CRC mortality in Missouri.^13^ However, a US study using county‐level characteristics to examine the association with CRC mortality reported that county‐level CRC screening rates were positively associated with CRC mortality. This difference between our findings and this US study may be explained by an ecological study design without considering the interference at individual‐level information. In addition to county‐level CRC screening rates, our findings also shown that those living in rural areas appeared to have 1.1‐fold increased risk of CRC death compared to those not living in rural areas regardless of covariate adjustments. Our findings agree with those of previous studies that have shown that rural residents have lower CRC survival compared to those living in non‐rural areas.^12^, ^26^, ^27^, ^28^, ^29^, ^30^

When exploring the impact of rurality, we observed that non‐rural patients living in intermediate and high‐screening areas were 9%–10% less likely to die from CRC in comparison with those living in low screening areas. Particularly, rurality has a significant negative effect on risk of CRC death in high‐screening areas (*p*‐value = 0.018). These findings suggest that county‐level rurality seems to be more important for explaining the disparities of CRC survival among patients living in high‐screening areas despite patients living in those areas potentially having more sources of care.^25^ Our findings also suggest CRC screening rates do not independently explain disparities in the risk of CRC death between non‐rural and rural populations.^31^ Other factors likely play a role such as the frequency of contact with healthcare providers, differences in access to chemotherapy, and difficulty in obtaining aftercare in rural settings.^31^ More research examining mediators/modifiers of the association between CRC screening, rurality, and CRC survival could clarify these relationships.

A major strength of this study is that we included county‐level CRC screening rates and rurality, which may have potential for reflecting actual resources of communities, as well as individual‐level covariates, which may reflect on how communities' resources impact an individual's risk of CRC death in Georgia. The findings from our study suggest that county‐level rurality plays an important role in CRC survival, particularly for high CRC screening areas. In order to address disparities in CRC mortality in areas with lower CRC screening rates and rural areas, interventions are needed to improve the accessibility, timeliness, and quality of CRC treatment, and follow‐up care^32^ as well as the availability of CRC screening.^33^ For example, patient navigation is likely to be useful in helping to ensure that patients newly diagnosed with CRC receive timely and appropriate treatment.^34^ Other interventions, such as assisting with patient transportation, may also be helpful in rural areas.^8^

Despite its strengths, there were a few limitations that should be noted. First, we were unable to examine if county residents who had CRC screening were the same individuals diagnosed with or dying from CRC. We were also unable to examine whether county‐level CRC screening rates included the same respondents with multiple screening use due to a cross‐sectional design from the NHIS and BRFSS survey. These may either underestimate or overestimate how county‐level CRC screening rates impact on survival outcomes in Georgia. Further research should investigate this multi‐level relationship on CRC outcome in more detail. Second, although the study has accounted for many individual‐level information in analyses, the SEER database did not capture individual sociodemographic characteristics (e.g., income and education level), lifestyle risk factors (e.g., obesity, smoking, and alcohol consumption), and comorbidities.^35^, ^36^, ^37^, ^38^ Further, county‐level CRC screening rates may not fully reflect actual healthcare resources that are linked to CRC outcomes. Limited treatment options also play a critical role in diagnosis outcomes for CRC patients. Finally, systemic barriers to cancer screening for CRC were not available for investigation in this study. Factors, such as limited CRC screening options and follow‐up care, assistance with scheduling, and preparation instructions^16^ can also contribute to adverse CRC outcomes.

## 5 CONCLUSIONS

Findings from our study found that patients living in low CRC screening rates counties had lower 5‐year survival rates. Five‐year survival rates were also significantly lower when patients living in rural areas with high‐screening rates. Patients living in intermediate and high CRC screening areas were negatively associated with the risk of CRC death. Particularly, non‐rural patients living in intermediate and high CRC screening areas were 8%–9% less likely to die from CRC. Therefore, our study suggests that targeted CRC screening resources should be prioritized for rural communities and areas with low screening rates.

## AUTHOR CONTRIBUTIONS

**Meng‐Han Tsai:** Conceptualization (equal); data curation (equal); formal analysis (equal); methodology (equal); writing – original draft (equal); writing – review and editing (equal). **Steven S. Coughlin:** Methodology (equal); writing – original draft (equal); writing – review and editing (equal). **Jorge E. Cortes:** Supervision (equal); writing – review and editing (equal).

## FUNDING INFORMATION

This research was supported at least in part through the Georgia Cancer Center Paceline funding mechanism at Augusta University (principal investigator: Meng‐Han Tsai, MCGFD01050).

## CONFLICT OF INTEREST STATEMENT

The authors declare no potential conflict of interest.

## ETHICS STATEMENT

Data extracted for this study were publicly available and de‐identified, and thus considered exempt from IRB review at Augusta University.

## Supporting information


Figure S1.

## Data Availability

The datasets generated during the current study are available in the Surveillance, Epidemiology, and End Results Program (https://seer.cancer.gov/), the United States Department of Agriculture rural–urban continuum codes (https://www.ers.usda.gov/data‐products/rural‐urban‐continuum‐codes/), and the National Cancer Institute (NCI) small‐area estimates of screening behaviors (https://sae.cancer.gov/nhis‐brfss/) repository.
