# Supplementary material for: County‐level colorectal cancer screening rates on colorectal cancer survival in the state of Georgia: Does county‐level rurality matter?
Source: Cancer Med. 2024 Jan 2;13(1):e6830. doi: 10.1002/cam4.6830 (PMC10807605; doi:10.1002/cam4.6830)
Supplement: Supplementary file 1 — Figure S1. [file CAM4-13-e6830-s001.docx]

SEER 18: CRC patients, 1975-2016

(n=997,685)

Exclusion (n=246,981):

1. Under 18 years of age, n=675
2. Repeated diagnosis, n=44,809
3. Missing rural-urban information, n=14,109
4. CRC diagnosed after 2011 due to not enough follow-up time, n=177,707
5. Missing survival time, n=9,681

CRC patients aged 18+

(n=750,704)

Exclusion (n=728,544):

1. Non-Georgia information, n=689,736
2. Missing cancer sites, n=343
3. CRC diagnosed before 2004 or after 2010, n=35,154
4. Under 50 years of age, n=3,311

CRC patients aged 50+ in Georgia

(n=22,160)

**Supplementary Figure 1. Flowchart of eligible participants**

Abbreviation: SEER, Surveillance, Epidemiology, and End Results Program ;CRC, colorectal cancer.
